# Reply to Martetschläger, F.; Wahal, N. Comment on “Feuerriegel et al. Assessment of Acute Lesions of the Biceps Pulley in Patients with Traumatic Shoulder Dislocation Using MR Imaging. *Diagnostics* 2022, *12*, 2345”

**DOI:** 10.3390/diagnostics13010026

**Published:** 2022-12-22

**Authors:** Georg C. Feuerriegel, Jan Neumann, Markus Wurm

**Affiliations:** 1 Department of Radiology, Klinikum Rechts der Isar, School of Medicine, Technical University of Munich, 81675 Munich, Germany; 2 Department of Trauma Surgery, Klinikum Rechts der Isar, School of Medicine, Technical University of Munich, 81675 Munich, Germany

We would like to thank you for your kind letter and thoughtful comments [1]. We agree that, in our current study, lesions of the biceps pulley were defined by injury to the sGHL alone (superior gleno-humeral ligament), CHL alone (coraco-humeral ligament), or as a combination of both the sGHL and CHL, detected by multiplanar MRI (Materials and Methods, page 3, paragraph 2). Martetschläger et. al. published a classification system for pulley lesions that is based on arthroscopic results (as mentioned in the Discussion, page 7, paragraph 4), which is very detailed and probably the most accurate arthroscopic classifications system so far. Unfortunately, the resolution of standard 3T MRI is yet not detailed enough to distinguish the different parts of the medial and lateral sling as proposed by Martetschläger et al. However, with the current development of deep-learning based image enhancement algorithms, a future study aiming at the differentiation of the medial and lateral pulley sling would a valuable investigation and may lead to a more concurring diagnosis by means of MRI. We decided to include the classification of Habermeyer et al., cited in Table 2, as it comes closest to the grading system that we used in our study by means of MRI, including the assessment of concomitant injuries, e.g., to the supraspinatus and subscapularis tendon as well as to the long head of the biceps tendon. Habermeyer et al. did not mention the CHL in their classification, yet we did include the detected CHL lesions for completeness. The classification system for lesions of the biceps pulley used in this study was explained in detail in our materials and methods section and therefore should not lead to any confusion with respect to the results of our study. Overall, our current study did not assess the reliability of classification systems, but rather the occurrence of lesions of the biceps pulley in patients with traumatic shoulder dislocation assessed by means of MRI. We hope you will find our responses satisfactory and thank you once more for your comments.
